# How Do Comeback Korean Pop Performers Acquire Audience Empathetic Attachment and Sustained Loyalty? Parasocial Interactions Through Live Stream Shows

**DOI:** 10.3389/fpsyg.2022.865698

**Published:** 2022-03-28

**Authors:** Zhuang Ma, Linpei Song, Jue Zhou, Woonkian Chong, Wantong Xiong

**Affiliations:** ^1^International Business School, Chongqing Technology and Business University, Chongqing, China; ^2^School of Business Administration, Gachon University, Seongnam, South Korea; ^3^School of Arts, Chongqing University, Chongqing, China; ^4^SP Jain School of Global Management, Sydney, NSW, Australia; ^5^College of State Governance, Southwest University, Chongqing, China

**Keywords:** comeback KPop performers, empathetic attachment, sustained loyalty, parasocial interactions, live stream shows

## Abstract

Live stream platforms have transformed the production and consumption of music, allowing KPop music to expand globally. Successful KPop idols are contrasted with large numbers of retired KPop performers, some of whom live in undesirable conditions. Drawing on the attachment theory, loyalty theory, and parasocial interaction theory, this study focuses on a unique group, comeback KPop performers, to examine how they acquire empathetic attachment and sustained loyalty from audiences through live stream shows, and the antecedents (i.e., sustained attractiveness, nostalgic experience, and parasocial interactions) of these two variables. Answering these questions seems important because comeback KPop performers have to interact with audiences without the financial and marketing support from entertainment agencies. The structural equation modeling of 288 responses from 176 Chinese and 112 Korean KPop audiences confirmed that empathetic attachment and sustained loyalty are positively associated with audience purchase intentions; sustained attractiveness and parasocial interactions function as antecedents of these two factors. The findings shed light on the comeback KPop performers who co-create value with audiences through live stream platforms, with theoretical contributions to the three theories mentioned above and managerial suggestions to KPop entertainment agencies, comeback KPop performers, and managers of live stream platforms.

## Introduction

Live stream performances (Kim et al., 2020) have transformed the production, consumption, and means of dissemination and access for music (Naveed et al., 2017; Han and Yoon, 2018). One live music genre that has benefited from live streaming is Korean pop (KPop) music (Lee et al., 2021). KPop, a cultural component of Hallyu (Korean wave), is a music genre paired with fashionably dressed boy/girl bands performing choreographed dances (Han, 2017). Empowered by social media platforms, KPop has developed from local popular culture to a transnational cultural phenomenon, attracting around 100 million loyal audiences worldwide (Emily, 2018). During the COVID-19 pandemic lockdown in 2020, the consumption of KPop live streaming has allowed South Korea’s three leading KPop entertainment agencies (JYP, YG, and SM) to achieve sales of 2.64 billion USD (Haydn-an, 2020). While the COVID-19 pandemic has been controlled in some countries (e.g., China) with the increasing number of vaccinations, live stream concerts and festivals have persisted (Bruner, 2021).

Due to its global influence, KPop has drawn increasing academic attention (Lee et al., 2021). So far, the KPop music literature has investigated several key success factors for KPop music. Aini et al. (2021) conducted a literature review regarding entertainment agencies’ strategies, including recruiting and debuting system (Diari & Mahyuni, 2019), and the “idol farm system:” recruiting promising talents, training, debuting the prepared idols, promoting idols, and proving services to audiences. Lee et al. (2021) adopted text mining and qualitative method to identify individual-level factors, such as visual beauty, performers’ sincere communication with audiences, empathy with lyrics, and audience benefits. Kim S. et al. (2021) highlight the facilitating role of a specific social media platform, V Live, suggesting that video platforms are important channels for KPop performers to communicate directly with audiences through live videos. However, most of these studies on KPop shows have been descriptive and conceptual, with limited empirical evidence (e.g., Kim S. et al., 2021) regarding the impacts of identified factors on audience intention to purchase live streaming KPop shows.

We argue that those success factors may not apply to the comeback KPop performers, i.e., retired KPop performers who have passed their “heydays” in KPop entertainment agencies and retired but later decided to return to the various shows on social media platforms. For instance, Sunye debuted as the leader of JYP Entertainment’s Wonder Girls in 2007, left the group in 2012, and came back to the stage in 2018. The success of KPop groups has attracted large numbers of teenagers who idolize and follow these successful KPop performers’ footsteps by auditioning at well-known entertainment agencies. However, the intense market competition means even successful KPop performers may quickly become “obsolete” with the increasing number of young trainees. Contracts with entertainment agencies are often limited to a short time, an issue known as the “7-year curse.” Without the financial and marketing support from entertainment agencies, retired KPop group members (usually at the age of 30) often work as solo performers, music producers and entertainers, with some living in poor conditions and subject to precarity and suicides (Longenecker, 2019). Such situations have drawn critical social and academic concerns (Padget, 2017; Susilo, 2020; Boman, 2021). In response to such concerns, this study draws on attachment theory, loyalty theory, and parasocial interaction theory to address the gap regarding KPop audience attitudes toward comeback KPop performers’ live stream shows, knowing that those comeback performers may not have the same aesthetic appearance and performing skills than before. Investigating comeback KPop performers makes important theoretical sense regarding the mechanisms of audience sustainable empathy and loyalty to comeback performers by purchasing those performers’ live streaming shows and sheds light on the power of consumers to positively influence and morally change specific social issues (Schlaile et al., 2018) such as retired KPop performers’ wellbeing. Following the above discussion, we develop the following research question (RQ):

RQ: How do comeback KPop performers sustain audience attachment and audience loyalty through live stream shows?

By answering the research question, we aim to (1) identify the sources of sustainable audience attachment to comeback KPop performers during live stream shows, (2) identify the sources of sustainable audience loyalty to comeback KPop performers during live stream shows, and (3) examine the collective impacts of these factors on audience purchase intentions of comeback KPop performers’ live stream shows.

By addressing these research aims, this paper advances the live stream and KPop literature by investigating how audience perceptions (i.e., sustained attractiveness, nostalgic experience, and parasocial interactions) affect their attitudes (i.e., empathetic attachment and sustained loyalty), which further influence their purchase intentions. In doing so, we tested the attachment theory by placing it in a unique context, i.e., comeback KPop performers’ live stream shows; extended the loyalty theory by examining the time boundary of loyalty theory; and complemented the parasocial interaction theory by examining the effect of two-way parasocial interactions on consumer attitudes. To the best of our knowledge, this is one of few studies that investigated comeback KPop performers who no longer have the financial and marketing supports from KPop agencies. Studying these performers’ live stream shows sheds light on the gig economy literature where freelance professionals co-create value through digital platforms.

The rest of this paper is organized as follows: the next section reviews the literature and presents the hypotheses, followed by the “Materials and Methods” Section that provides the research approach, variable measurement, Sample and Data Collection. The “Results” Section presents the results of empirical analyses, followed by the “Discussion” Section that discusses the empirical results, and finally, the “Conclusion” Section that presents the theoretical implications, managerial implications, limitations, and future search perspectives.

## Theoretical Background and Hypotheses

### Comeback Korean Pop Performers’ Live Stream Shows

The music market is filled with intense competition, with a limited share of musicians (around 0.2%) achieving success (Lee et al., 2021). Despite the intense competition, KPop music has successfully expanded globally, with famous KPop groups such as Bangtan Boys (BTS) earning widespread popularity and generating over $3.54 billion in revenue each year (Keith, 2021). Previous studies have identified several factors for KPop groups’ expansion to nearby countries and then to the world: strategic role of Korean entertainment companies (Kim J. H. et al., 2021), KPop group members’ traits (Lee et al., 2021), and the facilitating role of social media platforms (Kim S. et al., 2021). The performance quality of KPop groups is determined by their singing and dancing skills, lyrics that reflect social concerns, concert satisfaction, and visual attractiveness (Lee et al., 2021).

The above-mentioned key success factors may not entirely apply to comeback KPop performers’ situation. In this study, we define comeback KPop performers as the KPop stars who quit their KPop groups and ended contracts with entertainment agencies due to various reasons (e.g., marriage, scandals, and military services), but later resumed performance. Examples of comeback KPop performers include: Sunye, who debuted as the leader of JYP Entertainment’s Wonder Girls in 2007, left the group in 2012, and came back to stage in 2018; and Shoo, a member of SES, married in 2010 and back to perform in 2017. While a limited number of comeback KPop performers still have the financial and marketing support from entertainment agencies, many have lost such support and work as self-employed performers. Live stream platforms such as YouTube, Netflix, Viki, and V Live empower KPop performers with advanced streaming and recommendation features (Kim, 2012; Kim J. H. et al., 2021). With these platforms, comeback KPop performers can release a preview of their live stream shows and invite audiences into their channels for interactions and consumption. The availability of live stream platforms provides KPop audiences with new choice options (e.g., comeback KPop performers’ live stream shows), especially during the COVID-19 lockdown. These choices could modify their cognitive attitudes toward specific products/services, and further influence their purchasing habits (Drugãu-Constantin, 2019; Mirica, 2019; Rydell and Kucera, 2021). In this study, we identified two cognitive attitudes (empathetic attachment and sustained loyalty) that the available choice of comeback KPop performers’ live stream shows has influenced.

### Empathetic Attachment to Comeback Korean Pop Performers

Attachment is an emotional bond from an individual toward another individual (Ladhari et al., 2020). A higher level of attachment can lead to a stronger feeling of affection, connection, or empathy with the presence of the other individual. Attachment includes a reservoir of feeling states, allowing individuals to make short-cut judgment, which further affects their choices (Jin et al., 2022). The KPop literature has explained how audience emotional attachments toward performers are developed through involvement (Lee et al., 2019). In this study, we define “empathetic attachment” (EA) as KPop audience attachment toward comeback KPop performers, whose live stream show could elicit audience empathies toward those performers’ current situations (e.g., reduced income and precarious living conditions). According to the attachment theory, the available and supportive others in times of need can help an individual to develop effective emotion regulation, mental health, and psychosocial functioning, thereby developing attachment toward the one who has offered help (Bowlby, 1980). In their heydays, KPop stars would provide audiences with inspiration, encouragement and reassurances through music lyrics, audience meetups, and charities, which could comfort audiences going through hard times, improve their psychological wellbeing, especially when comfort is hard to access elsewhere. Through live stream platforms, comeback KPop performers can easily access previous audiences through video clips and live shows, with the reminiscent videos eliciting audience memories and empathies toward comeback KPop performers. For instance, comeback KPop performers can serve as vloggers to share their lives after retiring from KPop bands and entertainment agencies and demonstrate their authentic aspects to draw more audience attachment. According to Bowlby (1980), attachment can connect one individual to another one across time and space with an enduring emotional bond (Li et al., 2021). Such attachment can abridge the distance between pop stars and audiences, converting audiences into enthusiastic consumers (Stever, 2011; Stever and Lawson, 2013), whose emotional bonds and states could affect their motivation and purchase intention (Bagozzi et al., 1999; Ladhari et al., 2020). During interactions with the attached comeback KPop performers, audiences could develop empathetic feelings, which lead to purchasing behavior. With live stream platforms enhancing audience EA, we predict that the level of REA can influence audience intention to pay for comeback KPop performers’ live stream shows. Therefore, the following hypothesis can be proposed:

H1. Empathetic attachment has a positive impact on audience purchase intention of a comeback KPop performer’s live stream show.

### Sustained Loyalty to Comeback Korean Pop Performers

Loyalty refers to an individual’s commitment to re-purchase the specific product or service of a specific brand in the future, regardless of the situational factors and marketing efforts of other brands (Oliver, 1999). According to loyalty theory, loyalty can be attitudinal, i.e., a consumer’s deeply held intention to reuse or rebuy (Oliver, 1999; Chaudhuri and Holbrook, 2001). KPop audiences can become loyal and active, and willing to engage themselves in events that greatly interest them (Kozinets, 2001); in an online context, audiences are likely to purchase the products or services related to their favorite performers repeatedly over a long time (Kim and Kim, 2017, 2020). KPop audiences’ loyalty is often built on personal connections with their favorite KPop performers. To maintain connections, entertainment agencies often organize events such as meetups and “fansigns,” where KPop performers meet audiences, answer questions, sign on albums, and sing songs or play games together (Kim, 2018). Unlike the ongoing KPop performers who rely on entertainment agencies to manage audience events, comeback KPop performers have to rely on live stream platforms to deliver the experiences that could lead to sustained audience loyalty, which in this study refers to as the continued support to specific comeback KPop performers. Those supports could include audience intentions to read the news and watch music shows related to their favorite comeback KPop performers and share the relevant information with friends and families. A KPop audience with sustained loyalty will frequently think about a specific performer and even share a personal bias in favor of that performer (Funk et al., 2000). According to Sinclair and Tinson (2017), the felt loyalty to a specific artist could motivate audiences to continuously purchase the relevant products or services to support the artist. In other words, sustained loyalty could also drive audiences to invest in the products (e.g., live stream channels) of comeback KPop performers as a form of continuous support. Therefore, the following hypothesis can be proposed:

H2. Sustained loyalty has a positive impact on audience purchase intention of a comeback KPop performer’s live stream show.

Live stream platforms function as value co-creation hubs where comeback KPop performers produce and promote their music, and their daily lives; and audiences participate in discussions, generate new ideas, and share comeback Kpop performers’ shows via social media. Studies on value co-creation through online platforms suggest that perceived values are critical for consumers’ attitudes to services (Graessley et al., 2019; Meilhan, 2019; Watson, 2021). The literature has suggested three important components (i.e., sustained attractiveness, nostalgic experience, and parasocial interaction) that KPop audiences may perceive as valuable from comeback KPop performers’ live stream shows.

### Sustained Attractiveness as an Antecedent of Audience Empathetic Attachment and Sustained Loyalty

On live stream platforms, performers create attractive content and share relevant information to effectively engage audiences (Hu et al., 2017). Physical attractiveness plays an important role in live stream shows, as audiences tend to agree with the opinion of attractive performers and are more willing to comply with their suggestions (Van de Sompel and Vermeir, 2016). KPop performers are designed by entertainment agencies to fit the local beauty standards to be thin and physically recognizable (Elfving-Hwang, 2018). Before debuts, KPop performers go through systematic training offered by entertaining agencies, including singing, dancing, performing, foreign languages, and interview skills (Messerlin and Shin, 2013). Moreover, entertainment agencies manage KPop performers’ diets, public image and personalities (Pratamasari, 2017). According to Meng et al. (2021), live stream performers can stimulate audience pleasure (i.e., the degree to which audiences feel good, happy, satisfied and pleased with a live stream show) and arousal (i.e., the degree to which audiences feel excited, stimulated, awakened, and active in a live stream show) (Russell, 1980). Lee et al. (2021) further found that audience attachment to BTS, a successful KPop group, is linked to the stories and songs performed; and their loyalty is linked to performers’ features such as “beautiful,” “talented,” and “outstanding visual beauty.”

Given the above literature, comeback KPop performers’ sustained attractiveness, i.e., the enduring personality, appearance, talents that audiences perceive as attractiveness during live stream shows (Ha and Lam, 2017) could also stimulate audience pleasure and arousal. For instance, Dia, former members of IOI and Uni. T, provides live stream shows on TVAfrica and YouTube, where she maintains friendly, warm, and harmonious connections with audiences, creating a contagious atmosphere. Moreover, comeback KPop performers often attend “Survival” TV shows to demonstrate their talents and recent conditions. We predict that such efforts, together with their previous videos (e.g., MVs) on social media, could demonstrate the sustained attractiveness that stimulates audience arousal during live shows. Drawing on Rydell and Kucera (2021), we can predict that audience perceptions of comeback KPop performers’ sustained attractiveness could generate positive attitudes. Therefore, the following hypotheses can be proposed:

H3. Comeback KPop performers’ sustained attractiveness has a positive impact on audience empathetic attachment.

H4. Comeback KPop performers’ sustained attractiveness has a positive impact on audience sustained loyalty.

### Nostalgic Experience as an Antecedent of Audience Empathetic Attachment and Sustained Loyalty

Nostalgia refers to a longing for the cherished past or a yearning for previous possessions or experiences at the individual or social (Holbrook, 1993; Reisenwitz et al., 2004). According to Schindler and Holbrook (2003), nostalgia denotes an individual’s preference as a positive attitude and a favorable affect toward specific objects (e.g., people and experiences) related to the individual’s past. Nostalgia can be evoked by songs and performers that convey symbolic meanings and social activities. For instance, the performers, costumes, atmosphere, music, and displayed items in KPop shows can associate audiences with specific memories of the past. Moreover, live stream platforms allow audiences to share nostalgic sentiments with others, thereby stimulating more audiences to join the show. Compared to current KPop performers, comeback KPop performers are more likely to stimulate nostalgic experiences through live stream shows. For instance, the romance immersed in KPop music and shows of comeback KPop performers often reflects previous experience, thus allowing audiences to develop meaning in their previous social experience and even imagine their compensatory role in those shows. In other words, comeback KPop performers’ live stream shows could free audiences from everyday social life (i.e., the current life) and recollect the forgotten fantasies in their younger selves (Lee and Ju, 2010). The emotions and relationships, together with the narrative and visual technique expressed in comeback KPop performers’ live stream shows could elicit audience sentiment of nostalgia.

A nostalgia-evoking show could leave audiences with impressions of the past, thus eliciting spiritual empathy and enhancing their favorable attitudes toward a specific person or experience (Zhou et al., 2012). In a comeback KPop performer’s live stream show could deliver the nostalgic experience that stimulates audiences’ familiar feelings of the past and give them comfort, which further leads to emotional attachment toward the performer (i.e., someone in his or her heydays when the audience was younger). Moreover, previous studies have confirmed that nostalgic experience could influence customers’ intentions to rebuy specific products or services, thus closely related to customer loyalty (Chen et al., 2014; Su et al., 2016; Gu et al., 2021). Drawing on the relevant literature, we predict that nostalgic experience delivered in comeback KPop performers’ live stream shows could stimulate audience resonance about previous experience regarding the audience’s feelings and sympathies toward comeback KPop performers (i.e., sympathetic affection). Additionally, such nostalgic experience could motivate audiences to repeatedly seek memorable feelings and thereby become more loyal to the comeback KPop shows. Therefore, the following hypotheses can be proposed:

H5. The nostalgic experience delivered in comeback KPop performers’ live stream shows has a positive impact on audience empathetic attachment.

H6. The nostalgic experience delivered in comeback KPop performers’ live stream shows has a positive impact on audience sustained loyalty.

### Parasocial Interaction as an Antecedent of Empathetic Attachment and Sustained Loyalty

Unlike on-site communications, audiences are more likely to maintain a parasocial interaction with their favorite performers through social media by following and subscribing to performers’ live stream channels and postings (Chambers, 2013). The social media platforms provide an environment where audiences and their favorite KPop performers maintain a virtual relationship, with audiences assuming that the personalities appearing on live stream shows are friends and imagining that they are part of each show (Kim S. et al., 2021). Parasocial interactions (e.g., pushing “like” buttons and endorsing postings) allow audiences to believe that they share similar ideas, values and interests with their favorite performers, thereby feeling more connected to the latter (Frederick et al., 2012). Several scholars have assumed that parasocial interactions between audiences and their favorite performers are imaginary and one-sided (Dibble et al., 2016; Lim et al., 2020), while recent studies remind that the social media platforms (e.g., live streaming platforms) have extended the interaction from the one-sided and passive mode into a two-way interaction mode where audiences could interact with their favorite performers (Kim S. et al., 2021). Some audiences report enhanced feelings of intimacy and closeness through parasocial relationships compared to the face-to-face meetings with favorite performers, since radio talk allows them to better express their attachments freely (Xiang et al., 2016; Kim S. et al., 2021).

The two-way parasocial interactions on live stream platforms are important for comeback KPop performers who need to restore the affection and loyalty that audiences used to have during their heydays. In particular, audiences are likely to constantly spend time watching comeback KPop performers’ shows on several live stream platforms, where they have more opportunities to develop close relationships with those performers. Previous studies have proved that the rich experience developed from parasocial interactions could motivate audiences to feel more emotionally attached to their favorite performers (Frederick et al., 2012; Xu et al., 2020; Kim S. et al., 2021). Indeed, live stream platforms enable audiences to interact with their favorite performers more efficiently than offline meetups (Kim S. et al., 2021), since these platforms extend the limits of text messages into videos and audios, thereby enhancing audience attachment (Ming and Leung, 2017). Moreover, parasocial interactions may elicit audiences’ high degrees of empathy toward their favorite performers (Frederick et al., 2012). Through live stream platforms, audiences are able to interact with comeback KPop performers regarding the shared experiences and the personal lives of those performers, thereby developing empathetic attachment. According to Lee et al. (2021), audiences in frequent parasocial interactions with their favorite performers are likely to engage in impulsive sponsorship (e.g., purchase products and send gifts), thereby maintaining sustained loyalty. This mechanism may also function during the interactions between audiences and comeback KPop performers. Therefore, the following hypotheses can be proposed:

H7. Parasocial interactions between audiences and comeback KPop performers through live stream platforms have a positive impact on audience empathetic attachment.

H8. Parasocial interactions between audiences and comeback KPop performers through live stream platforms have a positive impact on audience sustained loyalty.

**FIGURE 1:**
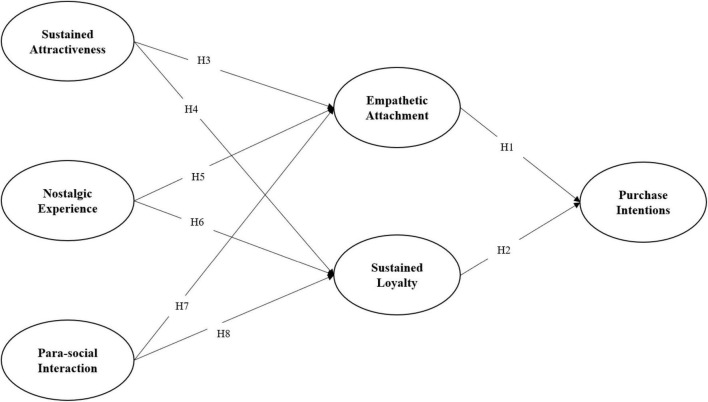
Conceptual framework.

We developed a theoretical framework based on the above hypotheses (see Figure 1), which were further tested with empirical data.

## Materials and Methods

### Research Approach

We adopted a quantitative strategy to analyze the impacts of empathetic affection, sustained loyalty, and their antecedents (i.e., sustained attractiveness, nostalgic experience, and parasocial interactions) on audience purchase intention of comeback KPop performers’ live stream shows. The deductive and cross-sectional approach allowed us to develop hypotheses based on established theories test them with empirical data (Wilson, 2014). We collected cross-sectional data, a common practice in consumer and psychology research (Hsiao and Tang, 2021; Molinillo et al., 2021). Specifically, we distributed the questionnaire survey to effectively access a large sample of the target population.

### Variable Measurement

We measured each variable item by adapting scales in the existing literature into our context, i.e., comeback KPop performers’ live stream shows. Those measurement items were chosen because several prior studies found these scales reliable (Yin et al., 2018).

#### Sustained Attractiveness

The measures for comeback KPop performers’ sustained attractiveness (SA) were adapted from “performer attractiveness” in Xu et al. (2020) for this study. The four items for this construct included (1) I think that the comeback KPop performer is talented in live stream show, (2) I think that the comeback KPop performer has an enjoyable live streaming style, (3) I think that the comeback KPop performer has an interesting personality, and (4) I think the comeback KPop performer has an appealing appearance. Comeback KPop performers’ sustained attractiveness was measured on a 5-point Likert scale with response options ranging from 1 to 5 (1 = “strongly disagree,” to 5 = “strongly agree”).

#### Nostalgic Experience

The six items for nostalgic experience (NE) were modified from “fantasies about past eras” in Merchant and Rose (2013), which relate to an individual’s longing for the lived past and thus suits this study. The six items for this construct included (1), I fantasized about the past when watching the comeback KPop performer’s live stream show, (2) The comeback KPop performer’s live stream show took me back in time, (3) When I saw the comeback KPop performer’s live stream show, I became lost in the period (s) shown, (4) I imagined I was participating in the story of the past shown in the comeback KPop performer’s live stream show, (5) The comeback KPop performer’s live stream show made me nostalgic for the period shown, and (6) After watching the comeback KPop performer’s live stream show, my imagination was like an avalanche: I thought of all different things about the past. We removed three items from the scale because they were not relevant to our context (i.e., comeback KPop performers live stream shows). Nostalgic experience was measured on a 5-point Likert scale with response options ranging from 1 to 5 (1 = “strongly disagree,” to 5 = “strongly agree”).

#### Parasocial Interaction

Parasocial interaction (PSI) was measured by adapting the four items from “parasocial interaction” in Xu et al. (2020). The four items included, (1) During the comeback KPop performer’s live stream show, I feel as though the streamer and I are friends, (2) When I am watching the comeback KPop performer’s live stream show, I feel a sense of “we-ness” (togetherness) with the performer, (3) I feel as though the comeback KPop performer cares about my responses during the live stream show, and (4) I feel the comeback KPop performer is like an old friend. Parasocial interaction was measured on a 5-point Likert scale with response options ranging from 1 to 5 (1 = “strongly disagree,” to 5 = “strongly agree”).

#### Empathetic Attachment

Empathetic attachment (EA) was measured by adapting the four items from “Product Attachment” in Cho et al. (2019). The four items included, (1) The comeback KPop performer is very dear to me, (2) The comeback KPop performer has special meaning to me, (3) I have an empathetic feeling about the comeback KPop performer, and (4) The life of comeback KPop performer has moved me. We removed one item from the scale because it overlapped with items in “Parasocial Interaction.” Empathetic attachment was measured on a 5-point Likert scale ranging from 1 to 5 (1 = “strongly disagree,” to 5 = “strongly agree”).

#### Sustained Loyalty

Sustained loyalty (SL) was measured by adapting the five items from “loyalty” in Yeh et al. (2020). The five items included, (1) I support this comeback KPop performer, (2) I am willing to subscribe to the paid live stream channels of this comeback KPop performer, (3) I am happy about this comeback KPop performer’s live stream show, (4) Next time, I will still pay for the live stream shows of this comeback KPop performer, and (5) I have decided to continuously pay for this comeback KPop performer’s shows. Sustained loyalty was measured on a 5-point Likert scale ranging from 1 to 5 (1 = “strongly disagree,” to 5 = “strongly agree”).

#### Purchase Intention

Purchase intention (PI) was adopted from “purchase intention” in Putrevu and Lord (1994). The five items included, (1) It is very likely that I will purchase the streaming products of this comeback KPop performer, (2) I will pay and download the streaming products of this comeback KPop performer, and (3) I will definitely pay for the new streaming products of this comeback KPop performer. Purchase intention was measured on a 5-point Likert scale ranging from 1 to 5 (1 = “strongly disagree,” to 5 = “strongly agree”).

#### Control Variables

In selecting variables to include as controls, we focused on those variables that could potentially be viewed as alternative explanations for audience purchase intentions. We controlled for audience age, gender, nationality, occupation, education, and spending on KPop products, which are widely accepted predictors of employee performance (Button et al., 1996; Ng and Feldman, 2010; Lim et al., 2020).

### Sample and Data Collection

Our survey objects were KPop audiences from China and South Korea. Specifically, we approached KPop dancing schools/clubs (whose members are familiar with KPop shows and active KPop audiences), five from China and three from South Korea. After obtaining permission, we sent an online questionnaire link to each respondent. In the questionnaire, we first introduced the research context where some retired KPop performers decided to come back to the stage through live stream shows. In Section “Introduction,” we asked each respondent to (1) confirm whether he or she was a loyal audience of any of the 100 disbanded KPop groups (Ranker, 2022) and (2) whether they would support those disbanded KPop performers (likely retired) if they came back to stage through live stream shows. Those who answered “No” in any of these two questions would be guided directly to the end of the questionnaire, suggesting an unusable survey.

The online questionnaire included 33 questions. In Section “Theoretical Background and Hypotheses” of the questionnaire, we included six questions related to each respondent’s demographic information, i.e., age, gender, nationality, occupation, education, and spending on KPop concerts. Those questions were set as control variables as they may affect the results of the hypothesized relationships. Section “Materials and Methods” is related to sustained attractiveness, nostalgic experience, parasocial interaction, empathetic attachment, sustained loyalty, and purchase intention. A total of 700 questionnaires were distributed to the respondents. Finally, 302 questionnaires were returned, out of which 288 were usable.

### Demographics

Table 1 presents the demographic information. Among the respondents, 61.1% were Chinese, and 38.9% were Korean; 26.4% were male, and 73.6% were female; 87.5% were 20–24 years old, 11.1% were 25–29 years old, and 1.4% were 30 or above years old; 74% were students, 22.2% were professional, and 3.8% were others; in terms of annual spending on Kpop concerts, 59.4% were less than 100$, 34.0% were 100–300$, 4.5% were 300–500$, 1.4% were 500–1000$, and 0.7% were more than 1000$.

**TABLE 1:** Demographics.

		Frequency	Percent
Nationality	China	176	61.1%
	Korea	112	38.9%
Gender	Male	76	26.4%
	Female	212	73.6%
Age	20–24	252	87.5%
	25–29	32	11.1%
	30 and above	4	1.4%
Occupation	Student	213	74.0%
	Professional	64	22.2%
	Others	11	3.8%
Spending	Less than 100$	171	59.4%
	100–300$	98	34.0%
	300–500$	13	4.5%
	500–1000$	4	1.4%
	More than 1000$	2	0.7%

## Results

Data was analyzed using quantitative techniques. We conducted a confirmatory factor analysis and examined convergent validity and discriminant validity. The reliability of each factor was examined through Cronbach’s Alpha. Structural equation modelling (SEM) was employed to analyze the model and test hypotheses using the Mplus 8.0 software package. Mplus is a structural equation modeling approach that considers the measurement model and the theoretical structural model simultaneously (Chin, 1998).

### Common Method Bias

The subjective measures of constructs were collected through questionnaires, thus possibly leading to the concern of common method bias. We addressed the common method bias issue in this study through research design and examined it thereafter through statistical analysis (Podsakoff et al., 2003). In the research design, we informed the respondents on the anonymity of their responses and that there were no correct or wrong answers. We then used Harman’s single-factor test (Podsakoff et al., 2003) method to test common method bias. We used all the variables in exploratory factor analysis, and the first factor only contributed 38.288% of the variance, indicating no dominant factor accounting for the variances of all constructs (Aulakh and Gencturk, 2000).

### Reliability and Validity

We examined reliability through Cronbach’s alpha. The Cronbach’s alpha for every construct is higher than 0.7, as shown in Table 2, indicating a high level of reliability. Confirmatory factor analysis is conducted to establish convergent validity. According to Fornell and Larcker (1981), convergent validity is established if all the factor loadings in the construct exceed 0.7, the average variance extracted (AVE) exceeds 0.5, and the composite reliability (CR) exceeds 0.7. Table 3 indicates that each factor loading in each construct meets the minimum requirement. Specifically, the standardized factor loading of each item is over 0.7, the CR of SA is 0.915, of NE is 0.931, of PSI is 0.909, of EA is 0.872, of SL is 0.936, and of PI is 0.870 (>0.7), and the AVE of SA is 0.730, of NE is 0.692, of PSI is 0.716, EA is 0.631, of SL is 0.746, and of PI is 0.690 (>0.5), indicating that each variable has good convergence validity.

**TABLE 2:** Results of discriminant validity and correlation.

	Mean	SD	SA	NE	PSI	EA	SL	PI
SA	3.63	0.92	**0.854**					
NE	3.48	1.05	0.412**	**0.832**				
PIS	3.86	1.04	0.467**	0.440**	**0.846**			
EA	3.83	1.00	0.365**	0.268**	0.361**	**0.795**		
SL	3.73	0.92	0.505**	0.339**	0.397**	0.427**	**0.864**	
PI	3.76	0.93	0.379**	0.343**	0.340**	0.351**	0.389**	**0.831**

**P < 0.01; SA, Sustained Attractiveness; NE, Nostalgic Experience; PSI, Para-social Interaction; EA, Empathetic Attachment; SL, Sustained Loyalty; PI, Purchase Intention; Square roots of AVEs are on the diagonal in parentheses; N = 288.

**TABLE 3:** Results of reliability and validity.

Variables (Sources)	Item	STD. Factor Loading	CR	AVE	Cronbach’s Alpha
SA (Xu et al., 2020)	I think that the comeback KPop performer is talented in live stream show. (SA1)	0.888	0.915	0.730	0.914
	I think that the comeback KPop performer has an enjoyable live streaming style. (SA2).	0.810			
	I think that the comeback KPop performer has an interesting personality. (SA3)	0.843			
	I think the comeback KPop performer has an appealing appearance. (SA4)	0.874			
NE (Merchant and Rose, 2013)	I fantasized about the past when watching the comeback KPop performer’s live stream show. (NE1)	0.779	0.931	0.692	0.930
	The comeback KPop performer’s live stream show took me back in time. (NE2)	0.900			
	When I saw the comeback KPop performer’s live stream show, I became lost in the period (s) shown. (NE3)	0.856			
	I imagined I was participating in the story of the past shown in the comeback KPop performer’s live stream show. (NE4)	0.798			
	The comeback KPop performer’s live stream show made me nostalgic for the period shown. (NE5)	0.832			
	After watching the comeback KPop performer’s live stream show, my imagination was like an avalanche. (NE6)	0.819			
PSI (Xu et al., 2020)	During the comeback KPop performer’s live stream show, I feel as though the streamer and I are friends. (PSI1)	0.772	0.909	0.716	0.907
	When I am watching the comeback KPop performer’s live stream show, I feel a sense of ‘we-ness’ (togetherness) with the performer. (PSI2)	0.821			
	I feel as though the comeback KPop performer cares about my responses during the live stream show. (PSI3)	0.839			
	I feel the comeback KPop performer is like an old friend. (PSI4)	0.943			
EA (Cho et al., 2019)	The comeback KPop performer is very dear to me. (EA1)	0.750	0.872	0.631	0.871
	The comeback KPop performer has special meaning to me. (EA2)	0.772			
	I have an empathetic feeling about the comeback KPop performer. (EA3)	0.827			
	The life of comeback KPop performer has moved me. (EA4)	0.826			
SL (Yeh et al., 2020)	I support this comeback KPop performer. (SL1)	0.832	0.936	0.746	0.931
	I am willing to subscribe to the paid live stream channels of this comeback KPop performer. (SL2)	0.811			
	I am happy about this comeback KPop performer’s live stream show. (SL3)	0.898			
	Next time, I will still pay for the live stream shows of this comeback KPop performer. (SL4)	0.920			
	I have decided to continuously pay for this comeback KPop performer’s shows. (SL5)	0.854			
PI (Putrevu and Lord, 1994)	It is very likely that I will purchase the streaming products of this comeback KPop performer. (PI1)	0.857	0.870	0.690	0.870
	I will pay and download the streaming products of this comeback KPop performer. (PI2)	0.820			
	I will definitely pay for the new streaming products of this comeback KPop performer. (PI3)	0.815			

*SA, Sustained Attractiveness; NE, Nostalgic Experience; PSI, Para-social Interaction; EA, Empathetic Attachment; SL, Sustained Loyalty; PI, Purchase Intention; N = 288.*

This study also examined discriminant validity following the approach suggested by Fornell and Larcker (1981). According to this approach, the AVE of each construct should be higher than the squared correlation between the constructs. Table 2 presents the correlation coefficient and square roots of AVEs, and square roots of AVEs of each construct is higher than the correlation coefficient. Square roots of AVEs are given in boldface along the diagonals. According to Table 2, discriminant validity is also established. Descriptive statistics are also presented in Table 2, indicating the mean values and the standard deviations.

We also adopted the comparison model to examine the discriminant validity (see Table 4). The six-factor model fitted the data well. According to Table 5, χ^2^/df = 1.31, Comparative fit index (CFI) = 0.984, Tucker–Lewis index (TLI) = 0.982, Root mean square error approximation (RMSEA) = 0.033, Standardized root mean square residual (SRMR) = 0.034, while the other factor models revealed a poor fit for the data. Therefore, the six-factor model demonstrated adequate reliability and validity.

**TABLE 4:** Results of comparison model.

Comparison Model	c^2^	df	c^2^/df	CFI	TLI	RMSEA	SRMR
Six-factor model	371.555	284	1.31	0.984	0.982	0.033	0.034
Five-factor model	823.291	289	2.85	0.904	0.892	0.080	0.076
Four-factor model	1546.669	293	5.28	0.774	0.749	0.122	0.127
Three-factor model	2151.4	296	7.27	0.665	0.632	0.148	0.133
Two-factor model	2979.793	298	10.00	0.516	0.472	0.177	0.129
One-factor model	3277.588	299	10.96	0.463	0.416	0.186	0.135

*Six-factor model: SA, NE, PSI, EA, SL, PI; Five-factor model: SA, NE, PSI, EA + SL, PI; Four-factor model: SA + NE, PSI, EA + SL, PI; Three-factor model: SA + NE + PSI, EA + SL, PI; Two-factor model: SA + NE + PSI + EA + SL, PI; One-factor model: SA + NE + PSI + EA + SL + PI; SA, Sustained Attractiveness; NE, Nostalgic Experience; PSI, Para-social Interaction; EA, Empathetic Attachment; SL, Sustained Loyalty; PI, Purchase Intention.*

**TABLE 5:** Results of model fit.

Model Fit	c^2^	df	c^2^/df	CFI	TLI	RMSEA	SRMR
Recommended value	–	–	<3	>0.9	>0.9	<0.08	<0.08
Measurement model	549.726	418	1.315	0.976	0.974	0.033	0.060

### Hypothesis Testing

To test the hypotheses, we used Mplus 8.0 to test the structural equations model. The overall model fit indicates that the model fits the data well. It can be seen from Table 5 that, CMIN/DF = 1.315 (<3), CFI, TLI all reach the standard value (>0.9), RMSEA is 0.033 (<0.08), SRMR is 0.060 (<0.08). Therefore, the model fit indices for the structural model were all at an acceptable level. Maximum likelihood estimates (MLE) for each parameter of the research model’s paths are illustrated in Table 6.

**TABLE 6:** Result of hypotheses testing.

Path		STD. Estimate	Estimate	S.E.	Est./S.E.	*P*-value
SA	EA	0.263	0.248	0.070	3.517	0.000
NE		0.070	0.061	0.063	0.979	0.326
PSI		0.238	0.231	0.073	3.154	0.002
SA	SL	0.412	0.428	0.070	6.125	0.000
NE		0.104	0.101	0.061	1.638	0.101
PSI		0.188	0.201	0.071	2.823	0.005
EA	PI	0.268	0.272	0.073	3.752	0.000
SL		0.336	0.310	0.064	4.831	0.000
GENDER		0.026	0.051	0.116	0.440	0.660
AGE		0.021	0.048	0.131	0.367	0.713
OCCUPATION		−0.031	−0.051	0.094	−0.540	0.590
SPENDING		0.003	0.004	0.071	0.058	0.954

*SA, Sustained Attractiveness; NE, Nostalgic Experience; PSI, Para-social Interaction; EA, Empathetic Attachment; SL, Sustained Loyalty; PI, Purchase Intention; N = 288.*

According to the results of the hypotheses testing, (1) EA has a significant positive impact on PI (β = 0.268, *p* < 0.05), suggesting that H1 is established; (2) SL has a significant positive impact on PI (β = 0.336, *p* < 0.05), suggesting that H2 is established; (3) SA has a significant positive impact on EA (β = 0.263, *p* < 0.05), suggesting that H3 is established; (4) SA has a significant positive impact on SL (β = 0.412, *p* < 0.05), suggesting that H4 is established; (5) NE has no significant impact on EA (β = 0.070, *p* > 0.05), suggesting that H5 is not established; (6) NE has no significant impact on SL (β = 0.104, *p* > 0.05), suggesting that H6 is not established; (7) PSI has a significant positive impact on EA (β = 0.238, *p* < 0.05), H7 is established; PSI has a significant positive impact on SL (β = 0.188, *p* < 0.05), H8 is established. Table 7 provides a summary of the hypothesis testing results.

**TABLE 7:** Hypothesis testing summary.

Hypotheses	Results
H1: Empathetic attachment has a positive impact on audience purchase intention of a comeback KPop performer’s live stream show.	Supported
H2: Sustained loyalty has a positive impact on audience purchase intention of a comeback KPop performer’s live stream show.	Supported
H3: Comeback KPop performers’ sustained attractiveness has a positive impact on audience empathetic attachment.	Supported
H4: Comeback KPop performers’ sustained attractiveness has a positive impact on audience sustained loyalty.	Supported
H5: The nostalgic experience delivered in comeback KPop performers’ live stream shows has a positive impact on audience empathetic attachment.	Rejected
H6: The nostalgic experience delivered in comeback KPop performers’ live stream shows has a positive impact on audience sustained loyalty.	Rejected
H7: Para-social interactions between audiences and comeback KPop performers through live stream platforms have a positive impact on audience empathetic attachment.	Supported
H8: Para-social interactions between audiences and comeback KPop performers through live stream platforms have a positive impact on audience sustained loyalty.	Supported

## Discussion

Live stream platforms have transformed the production and consumption of music (Han and Yoon, 2018), providing a hub for value co-creation between audiences and performers (Meilhan, 2019). These platforms provide audiences with new forms of access to services (i.e., music), shape their cognitive attitudes, and influence their purchase habits, especially in the global dissemination of KPop music (Lee et al., 2021). Unlike the previous studies that have conceptually explored the key success factors for ongoing KPop stars (Lee et al., 2021; Keith, 2021; Kim S. et al., 2021), this study focuses on a special cohort of KPop performers, i.e., comeback KPop performers. Specifically, we empirically examined how these comeback performers acquire favorable attitudes (i.e., empathetic attachment and sustained loyalty) from audiences through live stream shows. Our focus on comeback KPop performers sheds light on the KPop industry, which is filled with intense competition that renders KPop performers quickly become “obsolete.”

Our empirical results supported most of the research hypotheses regarding the impacts of comeback KPop performers’ sustained attractiveness (SA), nostalgic experience (NE) and parasocial interaction (PSI) delivered through comeback KPop performers’ live stream shows, audience empathetic attachment (EA), and audience sustained loyalty (SL) on audience purchase intention (PI) of comeback KPop performers’ live stream shows. Our results confirmed the positive influence of EA on PI, thereby concurring with Bowlby (1980) regarding the enduring nature of attachment; confirmed the positive influence of SL on PI, thus endorsing previous studies (Kim and Kim, 2017, 2020) regarding the audience loyalty in an online context. We further illustrated how comeback KPop performers acquire SL by co-creating value with audiences through live stream platforms without the support of entertainment agencies, thereby shedding light on the research about value-cocreation behavior in the gig economy (Meilhan, 2019).

We also examined the impacts of audience perceived values (SA, NE, and PSI) on their attitudes toward comeback KPop performers’ live stream shows. Specifically, we confirmed that SA could positively affect EA and SL; and that PSI can also positively affect EA and SL. In doing so, we agreed with previous studies regarding KPop performers’ physical attractiveness (Hu et al., 2017) and talents (Pratamasari, 2017), as well as the enhanced effects of intimacy and closeness achieved through parasocial interactions in live stream shows (Xiang et al., 2016; Kim S. et al., 2021). Our results further suggest that SA has a stronger effect on both EA and SL, compared to PSI. Surprisingly, we found no evidence regarding the impact of NE on EA and SL. These contradict the previous studies (Lee and Ju, 2010; Chen et al., 2014; Su et al., 2016; Gu et al., 2021) regarding the influence of nostalgia in eliciting audience empathy and favorable attitudes. Such results could be explained by the hedonic nature of KPop music and the transient nature of live stream shows, requiring comeback KPop performers to constantly update contents to elicit audience attachment and loyalty; or comeback KPop performers’ live stream shows failing to deliver nostalgic feelings to audiences (possibly due to a lack of technological or marketing resources).

## Conclusion

Drawing on the attachment theory, loyalty theory, and parasocial interaction theory, this study examined two attitudinal factors, i.e., empathetic attachment and sustained loyalty, regarding their impacts on audience purchase intention of comeback KPop performers’ live stream shows; and explored and verified the audience perceptions (i.e., sustained attractiveness, nostalgic experience, and parasocial interaction) to responsible for those two factors, thereby proposing and empirically revising a theoretical framework regarding the success factors influencing comeback KPop performers’ live stream shows.

### Theoretical Contributions

The theoretical contributions of this study are threefold. First, we tested the attachment theory (Bowlby, 1980; Ladhari et al., 2020) by placing audience attachment in a unique context, i.e., comeback KPop performers’ live stream shows. In particular, we examined whether those live streams shows could elicit empathies toward those performers’ current situations. We suggest that the stream shows and vlogs could remind audiences of the emotional and spiritual supports they received during those performers’ heydays, and help comeback KPop performers to restore audience attachment (i.e., empathetic attachment), which leads to purchase intentions. In doing so, we extend attachment studies (Bowlby, 1980; Li et al., 2021) by unraveling how comeback KPop performers can elicit empathies from audiences, thereby restoring audience attachment. Second, we extend the loyalty theory (Oliver, 1999; Chaudhuri and Holbrook, 2001) by examining the time boundary of KPop audience loyalty. While audience loyalty to their favorite KPop performers has been confirmed in previous studies (Kim and Kim, 2017, 2020), such loyalty is built on effective personal connections and may not always last over time. Unlike ongoing KPop performers who rely on entertainment agencies for such connections, comeback KPop performers need live stream platforms to maintain the connections and acquire sustained loyalty from audiences. We found that securing sustained audience loyalty require comeback KPop performers to demonstrate sustained attractiveness and deliver a pleasant experience during live stream shows. Third, we contributed to the parasocial interaction theory by complementing the one-sided audience-performer interactions where audiences develop wishful identities (Dibble et al., 2016; Lim et al., 2020) with a two-way-interaction perspective to fit the context of parasocial interaction on live stream platforms. We argue that the one-sided interactions may not be able to generate the experiences that allow comeback KPop performers to retore audience attachment and loyalty. Unlike the traditional media such as TV shows, live stream platforms enable two-way parasocial interactions where comeback KPop performers can maintain timely communications with audiences and upgrade the audiences’ wishful identification by sharing more personal experiences and listening to their voices, thereby enhancing their felt emotional attachment and maintaining sustained loyalty.

### Managerial Contributions

This study contributes suggestions to managers of KPop entertainment agencies, comeback KPop performers, and managers of live stream platforms. First, responsible KPop entertainment agencies should anticipate the short career life span of KPop performers, and provide training for KPop trainees to embrace innovation and technology, so that the retired KPop performers could seize career opportunities on a global scale in music and cultural industries worldwide. Moreover, entertainment agencies could integrate retired KPop performers into ecosystems such as live stream commerce (Lakhan et al., 2021). Second, comeback KPop performers should pay attention to the audience perceptions (i.e., sustained attractiveness and parasocial interaction) that could generate positive audience attitudes, thereby acquiring audience attachment and loyalty. Specifically, comeback KPop performers should continuously comply with local beauty standards, relate to their personalities and public image while demonstrating their talents on live stream platforms. Those performers are also advised to adopt virtual platforms to improve audience “stickiness” (i.e., visit duration to streamers’ live stream channels) by creating parasocial interactions that make audiences feel closer and more intimate to performers and thereby demonstrate sustained loyalty (Li et al., 2021). Third, managers of live stream platforms should be aware of the business values that comeback KPop performers can deliver and serve as a bridge between those performers and business needs (e.g., brand ambassadors and live stream commerce artists). Additionally, live stream platforms can provide technical training for retired KPop performers to better interact with audiences, thereby improving these firms’ public image.

### Limitations and Future Research Perspectives

Despite our efforts, several limitations exist in this study and suggest perspectives for future research. First, our data collection process might limit the generalizability of the results. We distributed the questionnaire to dancing school/club members of KPop lovers in China and South Korea. Future studies could adopt other methods (e.g., experiments and case studies) and triangulate primary data with secondary data to better examine the hypothesized relationships among the variables investigated in this study. Second, despite our efforts to control participants’ nationality, the Chinese culture shares some similarities with the Korean culture; this might be another restraint to our result generalizability. Future studies can examine whether the same results can be reported by KPop audiences from Western cultures. Third, this study mainly drew on the attachment theory, loyalty theory, and parasocial interaction theory to investigate the impact of empathic attachment and sustained loyalty, as well as their antecedents (i.e., sustained attractiveness, nostalgic experience, and parasocial interaction) on audience purchase intentions of comeback KPop performers’ live stream shows. Future studies could adopt other theories (e.g., social identity theory) to investigate the relationship. We did not adopt social identity theory because we believe that this theory is less compatible with the context of this study, i.e., comeback KPop performers (i.e., performers disconnected with KPop fandoms), rather than how the fandom identity of a specific KPop star affects KPop fan attitudes (Kim S. et al., 2021).

## Data Availability Statement

The raw data supporting the conclusions of this article will be made available by the authors, without undue reservation.

## Ethics Statement

The studies involving human participants were reviewed and approved by the Chongqing Technology and Business University. The patients/participants provided their written informed consent to participate in this study.

## Author Contributions

All authors listed have made a substantial, direct, and intellectual contribution to the work, and approved it for publication.

## Conflict of Interest

The authors declare that the research was conducted in the absence of any commercial or financial relationships that could be construed as a potential conflict of interest.

## Publisher’s Note

All claims expressed in this article are solely those of the authors and do not necessarily represent those of their affiliated organizations, or those of the publisher, the editors and the reviewers. Any product that may be evaluated in this article, or claim that may be made by its manufacturer, is not guaranteed or endorsed by the publisher.
